# Bilateral Basal Ganglia Calcification: Fahr's Disease

**DOI:** 10.7759/cureus.4797

**Published:** 2019-06-01

**Authors:** Hsein Wei Ooi, Chaozer Er, Ikram Hussain, Navin Kuthiah, Veeraraghavan Meyyur Aravamudan

**Affiliations:** 1 Miscellaneous, Ministry of Health Holdings, Singapore, SGP; 2 Internal Medicine, Woodlands Health Campus, Singapore, SGP; 3 General Medicine, Woodlands Health Campus, Singapore, SGP

**Keywords:** fahr syndrome, fahr disease

## Abstract

Fahr’s disease/syndrome is a condition defined as bilateral striato-pallido-dentate calcinosis, a neurodegenerative disease with radiological findings of symmetrical and bilateral idiopathic calcifications of the cerebellum, periventricular white matter, and basal ganglia. Clinical correlation with radiological and a calcium metabolism panel is crucial in differentiating between Fahr’s disease and Fahr’s syndrome. We describe a case that presented with the clinical feature of a cerebrovascular accident and had an incidental radiological finding of Fahr syndrome. The clinical features, laboratory investigations, and clinical management of Fahr's disease/syndrome will be discussed in detail in the article.

## Introduction

Fahr’s disease was described by Karl Theodor Fahr in 1930 as a rare familial (autosomal dominant) disorder that presented with idiopathic basal ganglia calcification, as seen in the neuroimaging study [1]. This condition is presented clinically with a broad range of neuropsychiatric symptoms and extrapyramidal disorders. A post-mortem examination revealed non-atherosclerotic vascular disease in the centrum semiovale and striatum [2-3]. Herein, we present the case of a male patient who presented with symptoms suggestive of a cerebrovascular accident and had computed tomography (CT) findings which were suggestive of Fahr's syndrome. The lab investigations also showed hypocalcaemia which was also a sign of Fahr's syndrome. This article will emphasize the radiological features, clinical features, diagnostic criteria, and management of Fahr's syndrome/Fahr's disease.

## Case presentation

The patient was a 77-year-old Chinese male who presented with the acute onset of symptomatic non-vertiginous giddiness (vomiting), nocturnal right wrist numbness, chronic progressive visual blurring, and left-sided hearing loss. However, there was no associated weakness or numbness of the extremities. The patient had a history of hypertension and hyperlipidaemia and had not been taking his antihypertensive agent, statins, or aspirin.

On physical examination, the patient was afebrile, hypertensive with a blood pressure reading of 191/90, a pulse rate of 82 beats per minute, and oxygen saturation of 100% on room air. No focal motor or sensory deficits were detected at the time of presentation. There were no demonstrable cerebellar signs. Results from the fundoscopic examination were unremarkable. No goitre was palpated. The cardiac and lung examination results were unremarkable.

Laboratory investigations revealed a hypocalcaemia level of 2.12 mmol/L (normal: 2.25 - 2.5 mmol/L) and serum phosphate level of 0.98 mmol/L (normal: 0.8 - 1.4 mmol/L), although a serum parathyroid level was not evaluated. The renal panel showed acute renal impairment with a serum creatine level of 105 umol/L (normal: 80 - 95 umol/L). The serum electrolytes levels were normal with a sodium of 141 umol/L (normal: 135 - 145 umol/L) and potassium of 3.9 umol/l (3.5 - 4.5 umol/L). There was an incidental note of vitamin D insufficiency of 29.5 ng/mL (normal: 40 - 59 ng/mL), subclinical hypothyroidism (free thyroxine (FT4) of 13.1 (7 - 15 mg/L)), and a thyroid-stimulating hormone (TSH) level of 5.88 (normal: 0.4 - 4.5 U/mL). The electrocardiogram (ECG) showed sinus rhythm and a normal QTc of 453 ms (normal: 451 - 470 ms). A low-density lipoprotein (LDL) of 5.06 umol/L (normal: < 3.4 umol/L), high-density lipoprotein (HDL) of 1.03 umol/L (normal: 1 - 1.5 umol/L), and triglyceride level of 1.83 umol/L (normal: < 2.3 umol/L) were noted in the screening lipid panel.

**Figure 1:**
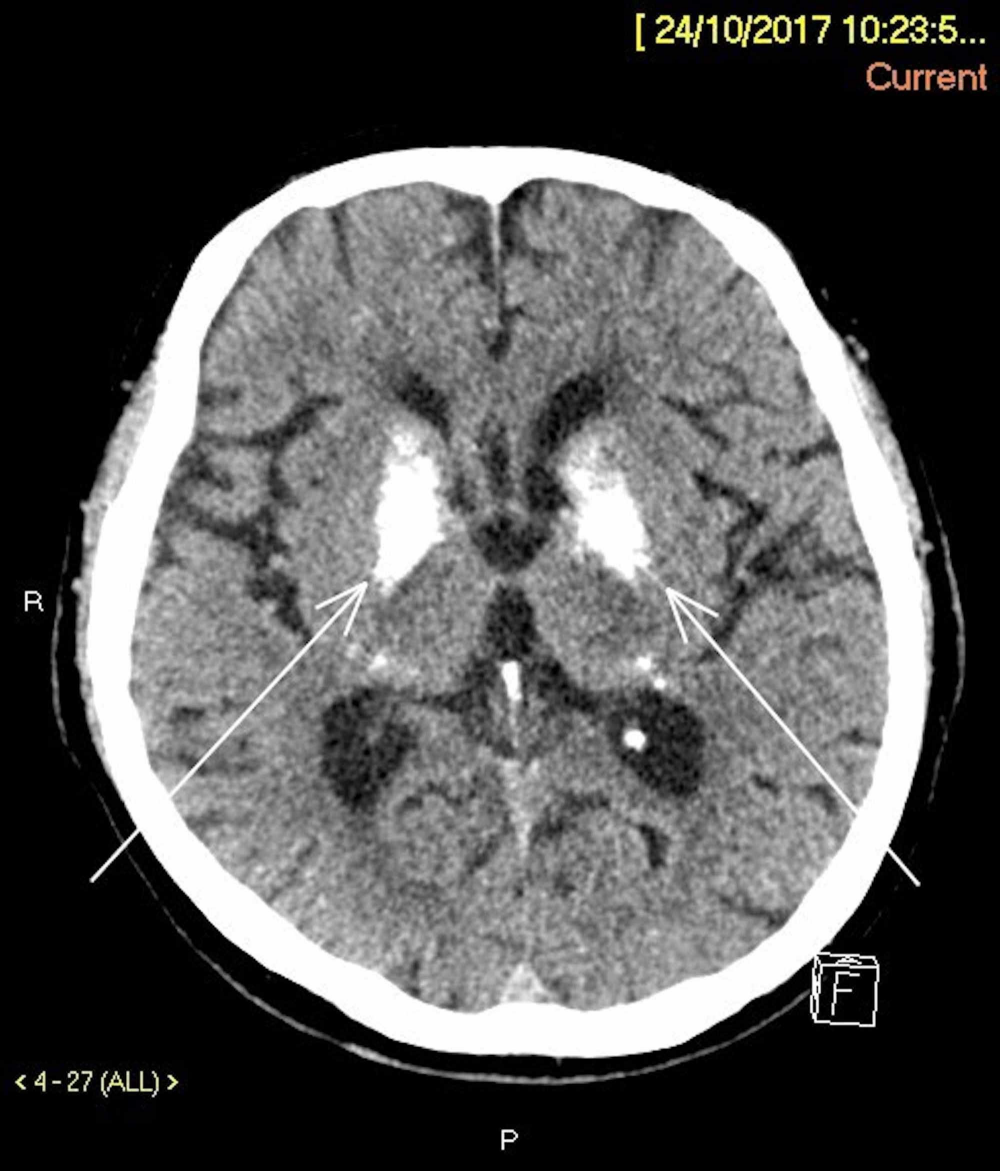
Axial Section of the Brain Shows Symmetrical Calcifications in the Corona Radiata (arrows).

CT imaging of the brain demonstrated confluent and asymmetrical calcification of the lentiform nuclei, thalami, corona radiata, and dentate nuclei (Figure 1). There was no evidence of acute intracranial haemorrhage or established territorial infarction. The patient’s symptoms resolved after an intramuscular administration of stemetil in the emergency department. Antihypertensive and statins were reinstituted in view of the clinical presentation of hypertension urgency, as well as hyperlipidaemia. The patient’s acute renal impairment resolved after intravenous and oral rehydration in the general ward. 

Neurology was consulted in view of the radiological findings demonstrated in the CT scan of the brain. The impression of the neurologist was possible Fahr syndrome which could still be incidental and the current clinical presentation could be due to accelerated hypertension. After optimal blood pressure control, he had a complete recovery and was discharged with advice on stroke prevention and blood pressure control.

## Discussion

Although both Fahr’s syndrome and Fahr’s disease resemble each other in terms of clinical signs and symptoms (e.g., neurological and psychiatric manifestations), there is still a clear distinction regarding the aetiology, location of calcifications, and treatment.

Table 1 is adapted from the diagnostic checklist by Perugula and Lippman to demonstrate the distinctions between these conditions [4].

**Table 1:** Diagnostic Features for Fahr’s Disease and Fahr's Syndrome Table adapted from the diagnostic checklist by Perugula and Lippman [4]

	Fahr’s Syndrome	Fahr’s Disease
Age of Onset	30 to 40 years old	40 to 60 years old
Genetic Traits	None	Autosomal dominant or recessive
Radiological Findings	Symmetrical and bilateral intracranial calcifications.	Coarse, progressive, bilateral and symmetrical striato-pallido-dentate calcifications.
Associated Conditions	Endocrinopathies: Idiopathic hypoparathyroidism secondary hypoparathyroidism, pseudo-hypothyroidism, hyperparathyroidism, or presence of any of the following conditions: Brucellosis infection (intrauterine or perinatal), neuroferritinopathy, tuberous sclerosis, mitochondrial myopathy, lipoid proteinosis	None
Treatment	Treatment directed to specific aetiology and adjunctive symptomatic treatment.	No specific remediation, only symptomatic treatment.

Prevalence

The exact prevalence of Fahr’s syndrome is uncertain; however, intracranial calcifications suggestive of Fahr’s syndrome are detected incidentally in approximately 0.3% to 1.2% of CT imaging of the brain [2].

Clinical features

Clinical features of both Fahr’s disease and Fahr’s syndrome are as listed in Table 2 below [5].

**Table 2:** Clinical Features of Fahr’s Disease and Fahr's Syndrome Pistacchi et al. [5]

Neurological	Psychiatric
Seizure	Cognitive impairment (dementia/delirium/confusion)
Movement disorder	Psychotic symptoms (hallucination/delusion)
Pyramidal signs/parkinsonism	Catatonia
Gait disorder	Irritability/aggression
Sensory changes	Personality disorder/personality change
Cerebellar abnormalities (vertigo)	Mood disorder
	Anxiety/obsessive behaviour

Complications

Other complications or clinical presentations include stroke, orthostatic hypotension, and syncope.

Diagnostic criteria

Diagnostic criteria for Fahr’s syndrome/disease are listed in Table 3 below [6-10].

**Table 3:** Diagnostic Criteria of Fahr’s Syndrome and Fahr's Disease Jaworski et al., Saleem et al., Pourshahid et al., Moskowitz et al., Manyam BV [6-10]

Diagnostic Criteria
Neuroimaging characterized by bilateral basal ganglia calcifications
Progressive neurological dysfunction that constitutes a variety of manifestations from motor disorder to neuropsychiatric presentation
The typical age of onset is thought to be around the fourth or fifth decades of life
In the absence of biochemical abnormalities or somatic features, another diagnosis has to be considered. For instance, mitochondrial disorders or metabolic conditions have to be excluded
Diagnosis of exclusion after evaluation for infectious, toxic, or traumatic causes
Presence of autosomal dominant familial inheritance disorder

Biochemical and haematological investigations

Baseline biochemical investigations are indicated to rule out other possible diagnoses (Table 4) [7-8].

**Table 4:** Laboratory Investigations in Diagnosing Fahr’s Disease Saleem et al. [7] CSF: cerebrospinal fluid

Types of Laboratory Investigations	Indication
Serum calcium, magnesium, phosphate, serum parathyroid hormones	To exclude hypocalcaemia, hypomagnesaemia, hyper- or hypoparathyroidism
Serum Vitamin D and calcitonin	To exclude vitamin D deficiency and secondary hypoparathyroidism.
Ellsworth-Howard test	To assess for hypoparathyroidism
Blood and urinary heavy metals level	To exclude heavy metal toxicity
CSF evaluation	To exclude infection and autoimmune aetiology

Genetic testing

Molecular genetic testing is indicated if there is a strong family history of autosomal dominant inheritance. SLC20A2 sequencing is the first test to be performed. If no identifiable mutation or deletion of SLC20A2, one must consider PDGFRB sequence analysis [7].

Type of genetic abnormalities

The exact aetiology of Fahr’s syndrome is still unclear. Genetic alteration at chromosome 14 has been suggested as a cause of this condition [3]. It is thought to be autosomal dominant in transmission. The 14q chromosome is most commonly affected in Fahr's syndrome [11-14]. 

Radiological findings

Radiological findings are usually detected incidentally in CT imaging for both Fahr’s disease and Fahr’s syndrome. These are the most important features as indicated in the diagnostic criteria. Bilateral calcifications of the basal ganglia, gangliocapsular region, and dentate nuclei are the classical radiological findings. Pathologically, calcifications occur in the vascular walls and in the perivascular spaces of arterioles, capillaries, and veins [7]. Laser spectroscopy demonstrated the presence of mucopolysaccharides and other minerals (zinc, phosphorus, chlorine, iron, aluminum, magnesium, and potassium). An MRI study has no significant role in imaging for these conditions. In the interest of MR imaging, the basal ganglia calcifications exhibit a low T2 signal and low to high T1 signal [15].

Management and treatment

Treatment for Fahr’s syndrome is tailored to the underlying associated conditions. Symptomatic treatment is most helpful. Symptomatic treatment can be pharmacological in nature. Table 5 below summarizes the drugs commonly used [16-18].

**Table 5:** Pharmacological Treatment for Each Symptom Lauterbach et al., Ramos et al., el Maghraoui et al. [16-18]

Symptoms	Treatment
Urinary Incontinence	Oxybutynin
Dystonia	Clonazepam
Dysparathyroidism-related movement disorders and seizures	Corticosteroids and Vitamin D3 supplementation
Depression and mood-related symptoms	Atypical antipsychotics
Seizures	Anti-epileptics

This case was slightly different from the published cases as the clinical presentation was non-specific, but the CT scan findings were suggestive of Fahr syndrome.

## Conclusions

Fahr’s disease and Fahr’s syndrome have a widespread clinical presentation with radiological findings of bilateral symmetrical basal ganglia and dentate nuclei calcifications. Therefore, it's essentially a diagnosis of exclusion after ruling out metabolic disorders. Treatment is tailored to symptom control for Fahr's disease and correction of underlying metabolic abnormalities. 
